# p97 complexes as signal integration hubs

**DOI:** 10.1186/1741-7007-10-48

**Published:** 2012-06-13

**Authors:** Hemmo Meyer

**Affiliations:** 1 Centre for Medical Biotechnology, Faculty of Biology, University of Duisburg-Essen, 45117 Essen, Germany

## Abstract

In the ubiquitin-proteasome system, a subset of ubiquitylated proteins requires the AAA+ ATPase p97 (also known as VCP or Cdc48) for extraction from membranes or protein complexes before delivery to the proteasome for degradation. Diverse ubiquitin adapters are known to link p97 to its client proteins, but two recent papers on the adapter protein UBXD7, including one by Bandau *et al*. in *BMC Biology*, suggest that rather than simply linking p97 to ubiquitylated proteins, this adapter may be essential to coordinate ubiquitylation and p97-mediated extraction of the proteasome substrate. These findings add to growing indications of richly diverse roles of adapters in p97-mediated signaling functions.

See research article: http://www.biomedcentral.com/1741-7007/10/36

## p97 - a simple view

**Figure 1:**
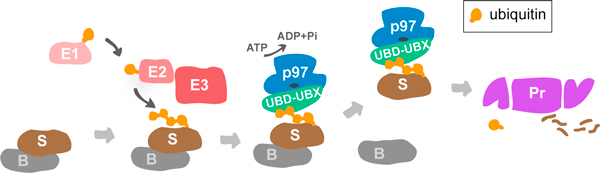
**A simple model for the function of p97**. A substrate protein (S) is ubiquitylated by a cascade of E1, E2 and the E3 ubiquitin ligase. If the substrate is tightly attached to a binding partner (B) or subcellular structure, p97 binds the substrate via a ubiquitin adapter containing a ubiquitin-binding domain (UBD) and a p97-binding UBX (ubiquitin regulatory X) or UBX-like (UBX-L) domain. Upon ATP hydrolysis, p97 extracts the substrate and delivers it to the proteasome (Pr) for degradation.

**Figure 2:**
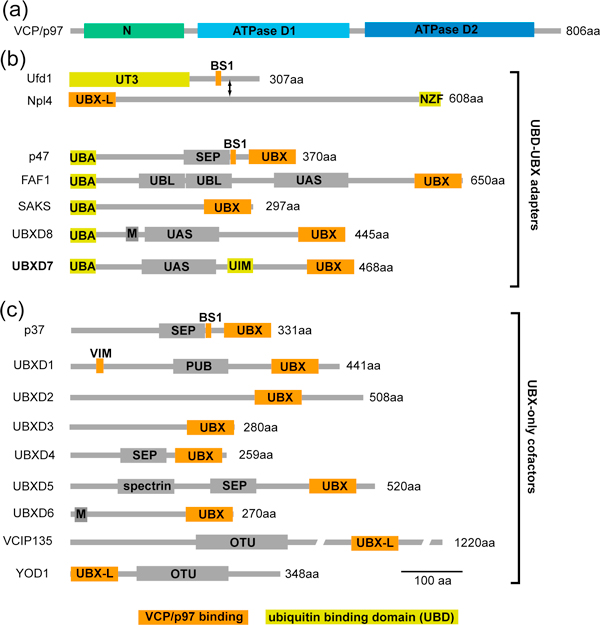
**Domain structure of p97 and UBX/UBX-L domain cofactors**. p97 cofactors are defined by p97-binding domains or motifs that directly interact with p97, and of which the UBX/UBX-like domain is the one that is found in the largest subset. Adapters are those cofactors that also contain a ubiquitin-binding domain (UBD), such as UBA, that links p97 to ubiquitylated substrates. **(a) **p97 contains two ATPase domains and an amino-terminal regulatory domain that binds cofactors and substrates. **(b) **Human UBD-UBX/UBX-L adapters comprise the Ufd1-Npl4 heterodimer and the five listed UBA-UBX proteins. **(c) **Other UBX and UBX-L cofactors are depicted. Not shown is a growing number of other cofactors that bind p97 directly through other domains. aa, amino acid; BS1, binding site-1; M, membrane anchor; NZF, Npl4 zinc finger; OTU, ovarian tumor deubiquitylatiing domain; PUB, peptide N-glysosidase/ubiquitin-associated) domain; SEP, Shp1-eyc-p47 domain; UAS, ubiquitin-associating domain; UBA, ubiquitin-associated domain; UBL, ubiquitin-like domain; UIM, ubiquitin-interaction motif; UT3, Ufd1 truncation 3 domain; VIM, VCP/p97 interaction motif.

The ubiquitin-proteasome system mediates degradation of misfolded or damaged proteins to ensure cellular protein homeostasis, and selectively removes regulatory proteins in critical signaling pathways. The key step is the posttranslational modification of the substrate with the small protein ubiquitin and extension to ubiquitin chains that serve as a signal for degradation by the proteasome. The ubiquitylation reaction is mediated by a cascade of enzymes, comprising the E1 ubiquitin-activating enzyme, an E2 conjugating enzyme and an E3 ligase that functions in the attachment of the ubiquitin to the substrate (Figure 1). Ubiquitin-binding shuttling factors then deliver the ubiquitylated substrates to the proteasome. The conserved hexameric AAA+ ATPase p97 has emerged as an important player during this step for a subset of client proteins [1,2]. Unlike other shuttle proteins, which simply bind both to ubiquitin chains and to components of the proteasome, p97 uses the energy of ATP hydrolysis to structurally remodel or unfold its clients, and is believed thus to help extract them from cellular structures, segregate them from binding partners or generate initial unfolded stretches to facilitate degradation by the proteasome. This is required for degradation of proteins associated with the endoplasmic reticulum, with the outer mitochondrial membrane, and with chromatin, and for some soluble proteins [1,2]. The interaction of the p97 ATPase with the ubiquitylated substrate is mediated by diverse ubiquitin adapters [1,2], which recognize both p97 and the ubiquitin chain on its client protein. Members of the largest family of such adapters are characterized by UBX and UBX-like domains, which assume a ubiquitin-fold and bind the amino-terminal domain of p97 [3]. They also contain ubiquitin binding domains (UBDs), including the UBA domain, that recognize the client. The domain structures of p97 and its UBD-UBX adapters are schematically illustrated in Figure 2. In a simple linear view, p97-adapter complexes bind substrates after ubiquitylation and then deliver them to the proteasome (Figure 1).

This simple view has been complicated, however, by a number of recent studies, including evidence from the Deshaies group [4] that p97 adapters can also link p97 to the E3 ligases that ubiquitylate the client proteins; and in two papers following up these studies, Alexandru and colleagues [5] and Deshaies and colleagues [6] report findings that add to increasing evidence for a central and far from simple role for p97 complexes in orchestrating the regulation of many cellular processes.

## A link to activated ligases

The starting point for the recent investigations was a mass spectrometry study by Alexandru and colleagues in Raymond Deshaies' group that revealed a far-reaching interaction network between p97 and a large number of E3 ligases, including cullin RING ligases (CRLs) [4]. Although for simplicity the E3 ligase in Figure 1 is schematically shown as a single entity, many E3 ligases are in fact complexes of several subunits with distinct functions. CRLs are among these multisubunit complexes, and consist of a cullin scaffold (CUL1, 2, 3, 4A, 4B or 5), a RING-domain protein that recruits an E2, and, except for CUL3-based CRLs, a cullin-specific adapter that in turn binds one of many substrate recruitment factors. In cullin-RING E3 ubiquitin ligases, the ubiquitin is directly transferred from the E2 enzyme to the substrate, the role of the cullin-RING being to recruit E2 and target protein and position them appropriately for ubiquitin transfer. Taking into account the different possible combinations of subunits, CRLs constitute up to 240 different ligase complexes that greatly extend the potential substrate spectrum of p97. Alexandru *et al*. [4] established the functional relevance of p97 binding to CRLs by showing that the degradation of the hypoxia-inducible factor HIF1α, a CUL2 substrate that is constitutively degraded in normoxic conditions, depends upon p97. Indeed, other CRL substrates have in the meantime been shown to require p97 for extraction: these include the mitotic kinase Aurora B and the polymerase II catalytic subunit Rpb1, which are ubiquitylated by CUL3, as well as the replication licensing factor Cdt1, which is targeted by CUL4A [1].

Importantly, while various p97-adapter complexes associate with different ubiquitin ligases, the UBXD7 adapter (also known as UBXN7, or in yeast, Ubx5) stands out because it is the only adapter to have been implicated in direct binding to CRLs. UBXD7 has now been shown, in parallel studies by the Alexandru and Deshaies groups [5,6], to bind directly to CRLs through a predicted ubiquitin-interacting motif (UIM) lying between its p97-interaction UBX domain and a UAS (ubiquitin-associating) domain of unknown function adjacent to its ubiquitin-binding UBA (ubiquitin-associated) domain (Figure 2). The UIM motif recognizes the ubiquitin-like small protein modifier NEDD8, whose dynamic conjugation to the cullin subunit of the CRL is required to activate its ligase activity [7]. Thus, mutation of the NEDD8 acceptor site on the cullin, or chemical inhibition of NEDD8 conjugation, both abolish binding of CRLs to UBXD7. And mutation of residues in the UIM domain of UBXD7, which are homologous to residues previously shown to be generally critical for ubiquitin binding by UIMs, also abolished binding of UBXD7 to neddylated CRLs. Interestingly, while transplantation of a UIM from another protein into UBXD7 only partially restored binding of the CRL, swapping NEDD8 for ubiquitin completely restored the interaction [6], suggesting that the UBXD7-UIM binds Nedd8 and ubiquitin, but that this is not generally true for other UIMs. However, while the UIM and NEDD8 were both required for UBXD7 binding to the CRL, they were not sufficient, suggesting that flanking residues of the UIM make essential contacts with other elements of the CRL complex. This is consistent with the observation that although the NEDD8 unit is identical in all CRLs, UBXD7 binds CUL2- and CUL4-based CRLs preferentially over CUL1 and CUL3 [6].

## A functional link to CRLs?

What might be the functional significance of UBXD7 binding to neddylated CRLs? In the case of the Cul3 substrate Rpb1 in yeast, the UBXD7 orthologue Ubx5 (and specifically its UIM) is required together with p97 for efficient degradation [6]. This suggests that linking substrate ubiquitylation and p97-mediated extraction may be important for efficient degradation of some substrates. Surprisingly, however, in the case of the CUL2 substrate HIF1α, p97 is required, but UBXD7 is not. On the contrary, Alexandru and colleagues found that UBXD7 depletion in cells accelerates HIF1α degradation [5]. Consistent with this, overexpression of wild-type UBXD7 inhibits HIF1α ubiquitylation, an effect that can be shown to be dependent on the presence of the UIM [5]. These observations suggest that UBXD7 acts as an antagonist of substrate ubiquitylation and degradation. This would not be surprising given the pivotal role of the UBXD7 docking site on CRL in the mechanism of CRL activation by neddylation, which involves NEDD8 and adjacent regions. NEDD8 modification induces conformational changes in the CRL that position the E2 in the vicinity of the substrate and thus promote ubiquitin transfer [7]. Indeed, Deshaies and colleagues mention that UBXD7 binding interferes with recruitment of the E2 Cdc34 to the CUL1 SCF^β-TrCP ^ligase and reduces substrate ubiquitylation [6]. Moreover, proper CRL activity requires NEDD8 to cycle on and off the cullin, while UBXD7 binding locks the CRL in the neddylated form. The function of a factor affecting such delicately balanced properties may be difficult to interpret because effects may be diverse depending on the experimental conditions.

Alexandru and colleagues [5] propose that inhibition of the ligase may indeed be the function of UBXD7 so that it reduces processivity of ubiquitylation and induces the generation of shorter ubiquitin chains. The short chains may specifically target the substrate to p97-mediated delivery to the proteasome, rather than to delivery pathways involving other adapters, such as Rad23, that tend to bind long chains. In contrast, Deshaies and colleagues [6] propose that UBXD7 may rather act as a sensor. Under normal conditions, the cycling of Nedd8 conjugation and de-conjugation may be too fast to allow UBXD7 binding. However, if the ligase encounters a substrate that is too tightly attached to a subcellular structure or binding partner and ubiquitylation and degradation is coupled, the ligase may stall. This would allow UBXD7 to bind to NEDD8 and then to recruit p97 for substrate extraction. This would introduce an element of specificity and explain why only certain substrates of CRL require p97-UBXD7. What it does not explain is why yet another subset of substrates of CRL requires p97 but not UBXD7, as is the case with chromatin-bound Cdt1, the replication licensing factor, which is targeted by CUL4A [6]. Clearly, further experimental work will be required to test the proposed models and address many more questions regarding UBXD7. In the meantime, it is clear that UBXD7 plays an interesting part in coordinating substrate ubiquitylation with p97-mediated extraction.

## Functional versatility of p97 complexes

**Figure 3:**
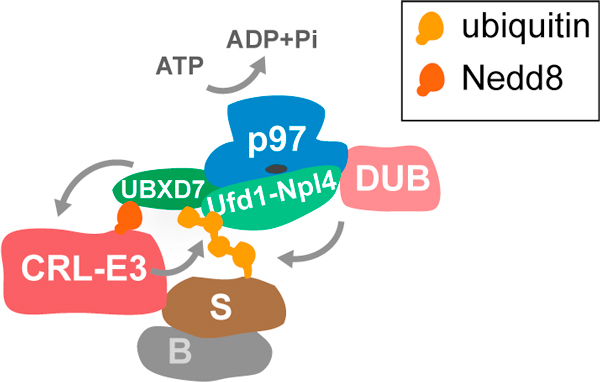
**A more detailed view of a p97-complex**. p97 binds the ubiquitylated substrate (S) through the Ufd1-Npl4 adapter to extract it from a binding partner (B). In addition, p97 is linked to the neddylated CRL-E3 ubiquitin ligase through the UBXD7 adapter, which binds Nedd8 via its UIM and possibly coordinates extraction with upstream ubiquitylation. p97 can also bind one of several deubiquitylating enzymes (DUB) that may edit the ubiquitin chains to control the downstream fate of the substrate.

The new insights into UBXD7 function add to accumulating evidence that p97-adapter complexes are more multifaceted and their function is more complex than in a simple linear model, where the adapters' sole role is to link the ubiquitylated client to p97 ATPase (Figure 3). One indicator of this complexity is that p97 can associate with several cofactors at a time. An emerging model suggests that a subset of cofactors including Ufd1-Npl4 or p47 constitute major adapters that can define core complexes and may even govern association of additional cofactors specific for particular pathways. One class of additional cofactors comprises targeting factors that recruit p97 to a specific subcellular location. A good example is the membrane protein UBXD8 (also called FAF2, or Ubx2 in yeast) that recruits p97-Ufd1-Npl4 to function in endoplasmic reticulum-associated degradation [2].

This cofactor diversity also entails complexity in ubiquitin binding. Cofactors including UBXD8, like UBXD7, themselves contain ubiquitin-binding UBA domains that work in addition to the UBDs in the p97-Ufd1-Npl4 core complex that they associate with. Conversely, there are core complexes with adapters that do not contain apparent UBDs: p97-p37 and p97-UBXD1 are such complexes [1]. It is unclear yet if these core complexes contain additional ubiquitin adapters or whether in these cases ubiquitin binding is mediated directly by p97, which can bind ubiquitin itself, albeit weakly.

An important class of cofactors with clear functional implications are the downstream processing factors that include a growing number of deubiquitylating enzymes [2]. They can either remove ubiquitin chains altogether to recycle the client protein by preventing its degradation, or conversely, they can facilitate substrate degradation by editing the ubiquitin chains to make them appropriate for proteasomal targeting. This suggests that p97 also acts as a relay point in ubiquitin-dependent processes that determines the fate of extracted substrates [8].

Further important implications for functional versatility can be drawn from the recognition that UBXD7 binds not only ubiquitin but also the ubiquitin-like modifier NEDD8. This raises the possibility that adapters may interact also with other ubiquitin-like modifiers, thus increasing the substrate spectrum of p97 and even further establishing it as a decoding and integration hub in far reaching signaling networks. Examples are the binding of the p47 adapter orthologue Shp1 to the ubiquitin-like modifier Atg8 during autophagy, or the association of p97 and Ufd1 with the SUMO system in yeast [9,10]. Lastly, several cofactors contain discrete but functionally poorly defined domains, including the UAS domain, which are likely to harbor additional functions that will add to the complexity of the system.

p97 is now established as a key element of ubiquitin-regulated processes. Clearly, ubiquitin adapters link p97 to ubiquitylated clients and thus position its function downstream of ubiquitination. However, it is becoming obvious that there is more to p97 complexes than mere substrate binding and extraction and that they constitute signal integration hubs. Much work lies ahead to understand the individual functions of the adapters, how p97 complexes modulate and coordinate events upstream and downstream of p97-mediated extraction and how this is embedded in complex signaling networks.
